# Validating chlorophyll-*a* concentrations in the Lagos Lagoon using remote sensing extraction and laboratory fluorometric methods

**DOI:** 10.1016/j.mex.2018.09.014

**Published:** 2018-09-29

**Authors:** A.O. Ayeni, T.A. Adesalu

**Affiliations:** a Department of Geography, University of Lagos, Lagos, Nigeria; b Department of Botany, University of Lagos, Lagos, Nigeria

**Keywords:** Remote Sensing extraction and laboratory fluorometric methods, Methods, Chlorophyll-*a*, Bio-optical models, Remote sensing, Satellite imagery

## Abstract

Remote sensing data is a viable alternative for mapping pigment concentrations in water body, and consequently, the trophic. Chlorophyll-*a (Chl-a*) is present in all phytoplankton species. This study therefore uses laboratory fluorometric and remote sensing extraction methods for assessing chlorophyll-*a* concentration in the Lagos Lagoon. The ﬂuorometer was calibrated with a commercially available chlorophyll-*a* standard before used in the laboratory to estimates chlorophyll-*a* concentration. Landsat 7 (ETM+) and Landsat 8 (OLI) were acquired for the remote sensing method. The Landsat data were first geometrically rectified. Then brightness values were converted to reflectance through the radiometric correction process. For the regression models, logarithmically transformed chlorophyll-*a* was used as the dependent variable. Single bands, band ratios and logarithmically transformed band ratios were the independent variables. R2 values were computed and evaluated.

• Chlorophyll-a contributes to productive water bodies
• laboratory fluorometric and remote sensing extraction methods
• Landsat data acquired for the remote sensing method

## Graphical abstract

**Figure:**
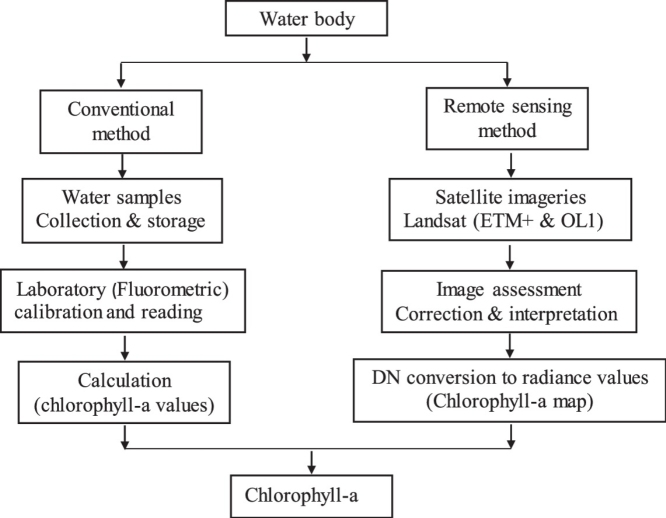

**Specifications Table**

**Table:** 

**Subject Area**	Environmental Geography and Biological Sciences
**More specific subject area:**	Environmental Hydrology for Water Security
**Method name:**	Remote Sensing extraction and laboratory fluorometric methods
**Name and reference of original method**	Ostrowska, M. (1990): Fluorescence “in situ” method for the determination of chlorophyll-a concentration in sea, OCEANOLOGIA, 29: 175 – 202 Gitelson, A. A., Y. Z. Yacobi, A. Karnieli, and N. Kress (1996): Reflectance spectra of polluted marine waters in Haifa Bay, Southeastern Mediterranean: features and application for remote estimation of chlorophyll concentration. Israel Journal of Earth Science, 45: 127–136
**Resource availability**	

## Method Details

Optical satellite datasets have been used to detect freshwater systems for decades however traditionally, satellite remote sensing of freshwater systems has been limited by sensor technology as well as its current and past missions have not provided the measurement resolutions needed to fully resolve freshwater ecosystem properties and processes [1]. Nevertheless, integration of earth observation products derived from satellite imageries that may improve water quality monitoring is one of the feasible methods [[2], [3], [4]]. Several studies methods have demonstrated the relationship between optical properties (reflectance) of water to other water parameters’ properties vis-a-vis suspended sediments, chlorophyll concentrations, dissolved organic matter, pigment load, temperature, Secchi disc depth and other laboratory based water quality [[5], [6], [7], [8]]. Satellites sensors can measure the amount of solar radiation at various wavelengths reflected by surface water, which can be compared to water quality parameters for instance, total suspended solids which constitutes an alternative means of estimating water quality [9,10]. Remote sensing therefore, offers a credible means of estimating water quality measurement. In a comparative study to assess the ability of satellite based sensors to monitor suspended sediment concentration, Secchi disc depth, and turbidity, it was discovered that predictions based on optical measures of water quality are slightly better when using earth observation data [11]. Apart from extremely demanding time and capital investments of traditional methods, its monitoring also requires sequential laboratory and unreliable in situ measurements and analysis [12].

It is on the aforementioned basis that this study established that both laboratory and satellite extraction methods have their merit and demerit.

## Methods

### Laboratory method

#### Sample collection and storage

**Fig. 1:**
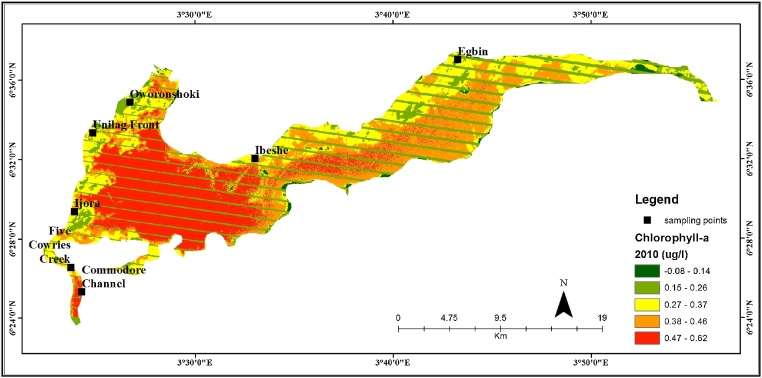
Estimated chlorophyll-*a* distribution in the Lagos Lagoon, 2010.

**Fig. 2:**
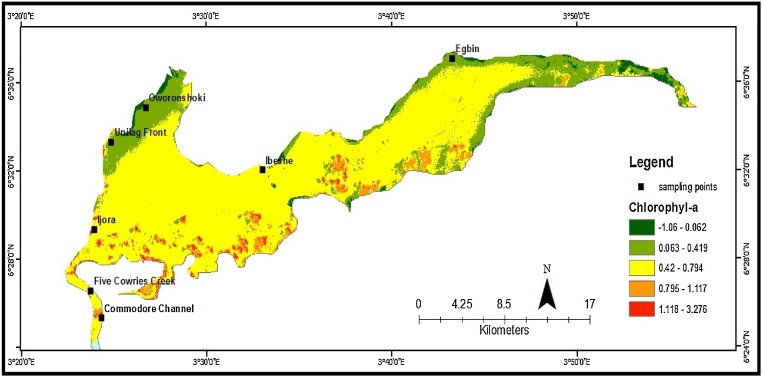
Estimated chlorophyll-*a* distribution in the Lagos Lagoon, 2015.

The samples points comprise of 7 locations points across Lagos lagoon. The locations include Ibeshe, Egbin, Oworoshoki, University of Lagos-UNILAG front, Ijora, Five Cowries Creek, and Commodore Channel (Table 1, Fig. 1, Fig. 2). The water samples were collected into clean polyethylene bottles. Water sample measured 200 ml was filtered through a 0.45 μm fibre membrane filter, after which the residue on the filter was transferred to a tissue blender, covered with 3 ml of 90% aqueous acetone and macerated for 1 min. the sample was then transferred to a centrifuge tube, capped and allowed to stand for 2 h in the dark at 4 °C (in a refrigerator). Samples were ﬁltered through 47 mm GF/F ﬁlters using polycarbonate in-line ﬁlters (Gelman) and a vacuum of less than 100 mm Hg. Filters are folded in half twice and wrapped in aluminum foil, labeled, and stored in refrigerator until ready for analysis. For ﬂuorometric analysis, we used 25 mm GF/F ﬁlters.

**Table 1:** Landsat data and Laboratory estimation of Chl-*a*.

Locations’ information			Landsat Imageries (μg/l)		Laboratory (μg/l)
Latitude	Longitude	Location	2010	2015	2015
6°25′14.5″	3°24′25.7′	Commodore Channel	0.48	0.25	0.32
6°26′17.4″	3°23′48.0″	Five Cowries Creek	0.32	0.24	0.44
6°27′54.0’’	3°22′37.3″	Ijora	0.32	0.23	22
6°30′37.5’’	3°24′14.1″	Unilag Water Front	0.32	0.20	0.014
6°32′54.0’’	3°24′24.6″	Oworonshoki	0.21	0.19	0.02
6°′32′48.9’’	3°28′36.1″	Ibeshe	0.32	0.23	0.16
6o25′37.8’’	3°35′55.1	Egbin	0.29	0.21	0.13

After removing the samples from refrigerator, the pigments are extracted by placing the ﬁlters in 5.0 ml 100% acetone. For 47 mm GF/F ﬁlters, 0.8 ml of water is retained adjusting the ﬁnal extraction solution to 86% acetone and the ﬁnal extraction volume to 5.8 ml. The samples are covered with Paraﬁlm to reduce evaporation, sonicated (0 °C, subdued light) and allowed to extract for 4 h in the dark at −20 °C. Following extraction, samples are vortexed, ﬁlters are pressed to the bottom of the tube with a stainless-steel spatula and spun down in a centrifuge for 5 min to remove cellular debris. For ﬂuorometric analysis (not HPLC), decantation can replace centrifuging.

Chlorophyll-*a*, ﬂuoresce in the red wavelengths after extraction in acetone are excited by blue wavelengths of light. The ﬂuorometer stimulates the extracted sample with a broadband blue light and the resulting ﬂuorescence in the red is detected by a photomultiplier. The signiﬁcant ﬂuorescence by phaeopigments is corrected for by acidifying the sample which converts all of the chlorophyll *a* to phaeopigments. By applying a measured conversion for the relative strength of chlorophyll and phaeopigment ﬂuorescence, the values were therefore used to calculate the chlorophyll-*a* concentrations.

Apparatus:

Filtration system and Whatman GF/F ﬁlters

Liquid nitrogen and freezer for storage and extraction

Glass centrifuge tubes for extraction, 15 ml

Turner ﬂuorometer, ﬁtted with a red sensitive photomultiplier, a blue lamp, 5–60 blue ﬁlter and 2–64 red ﬁlter.

Reagents

100% acetone

90% acetone

M HCl (100 ml HCl in 900 ml de-ionized water)

### Laboratory estimation of chlorophyll-*a*

For laboratory assessment, the ﬂuorometer was calibrated with a commercially available chlorophyll-*a* standard before used in the laboratory [[13], [14], [15], [16],^5^]. The standard is dissolved in 90% acetone for at least 2 h and its concentration (mg l^−1^) is calculated spectrophotometrically as follows:

(1) Chl-a = [(Amax – A_*750*_ _nm_)/E*l] *(1000 mg/1 g)

where:

*A*_max_ is absorption maximum (664 nm)

*A*_750_ _nm_ is absorbance at 750 nm to correct for light scattering

*E* is extinction coefﬁcient for chl-*a* in 90% acetone at 664 nm (87.67 L g^−1^ cm^−1^)

*l* is cuvette path length (cm).

From the standard, a minimum of ﬁve dilutions are prepared for each door. Fluorometer readings are taken before and after acidiﬁcation with 2 drops 1.2 M HCl. Thereafter, linear calibration factor (*K_x_*) are calculated for each door (x) as the slope of the unacidiﬁed ﬂuorometric reading vs. chlorophyll-*a* concentration calculated spectrophotometrically. The acidiﬁcation coefﬁcient (*F_m_*) was calculated by averaging the ratio of the unacidiﬁed and acidiﬁed readings (*F*_o_/*F*_a_) of pure chlorophyll-*a*. Samples are read using a door setting that produces a dial reading between 30 and 100. The ﬂuorometer is zeroed with 90% acetone each time the door setting is changed.

Chlorophyll-*a* was determined using a Fluorometer equipped with filters for light emission and excitation [5,15,17,18]. Thereafter, it was centrifuged at 5000 rpm for 20 min. and the supernatant was decanted. Volume left after decanting was noted. Different readings were taken from the Fluorometer (which had been pre-calibrated with 2, 5, 10 and 20 μg standard chlorophyll solutions) at ×1, ×3, ×10, and ×30 sensitivity settings and noted. The concentrations of chlorophyll-*a* for the samples were calculated using Eqs. (1) and (3):

(2) Chl (μg/l) = (*F_m_*/*F_m_* − 1)*[(*F_o_* − *F_a_*)*K_x_*(vol*_ex_*/vol*_filt_*)

(3) Phaeo (chl equiv.weights) = (*F_m_/F_m_* − 1)*[(F_m_*F_a_) − Fo]*K_x_* − vol_ex_

where:

*F_m_* is acidiﬁcation coefﬁcient (*Fo*/*Fa*) for pure Chl *a* (usually 2.2).

*F_o_* is reading before acidiﬁcation

*F_a_* is reading after acidiﬁcation

*K_x_* is the door factor from calibration calculations

vol*_ex_* is extraction volume

vol*_ﬁlt_* is sample volume.

## Remote sensing extraction method

### Image data processing

Landsat-7 ETM+ image is superior to its predecessors (e.g. Landsat -5), with significant improvement of on-flight geometric and 5% absolute radiometric calibration, and consist of improved panchromatic band with 15 m spatial resolution (band 8), Visible (reflected light) bands in the spectrum of blue, green, red, near-infrared (NIR), and mid-infrared (MIR) with 30 m spatial resolution (bands 1–5, 7), and a 60 m thermal infrared (band 6) spatial resolution (USGS, 2018).

Landsat 8 Operational Land Imager (OLI) and Thermal Infrared Sensor (TIRS) images consist of nine spectral bands with a spatial resolution of 30 m for Bands 1–7 and 9. The resolution for Band 8 (panchromatic) is 15 m. In addition, it also has two Thermal IR bands with a spatial resolution of 100 m (later resampled into 30 m). Since the spectral bands of Landsat ETM are very similar, this study used similar methods for 2007 and 2010 imageries. Using the image metadata, the radiometric calibration was conducted to convert digital numbers into top-of-atmosphere radiance Watanabe et al. [19,38]. The retrieval of the at-surface reflectance was accomplished using the Fast Line-of-sight Atmospheric Analysis of Spectral Hypercubes (FLAASH), an atmospheric correction module, implemented in the ENVI software. This tool adopted the MODerate resolution atmospheric TRANsmission (MODTRAN4), an atmospheric radioactive transfer code [20,19,[21], [22], [23]].

#### Image preprocessing and subset

The Landsat 7 and 8 images were imported into the ArcGIS environment and a shape file covering the Lagos lagoon was superimposed on the images and used to extract the Region of interest (ROI). The extracted images were then stretched using the histogram equalization technique and filtered to remove haze, cloud cover and noise using the Quick atmospheric correction tool in Envi 5.0 software [20,24].

Landsat ETM+ data pre-processing followed standard specification including radiometric and geometric calibration and terrain correction [25,26]; conversion from digital number to satellite reflectance (for six reflectance bands) or at satellite radiance temperature (the thermal band), and referencing to the National Albers equal-area map projection and resampling using cubic convolution to 30 m resolution. After initial pre-processing, tasselled-cap brightness, greenness, and wetness were derived using satellite reflectance-based coefficients [27,26].

### Estimation of chlorophyll-*a* using Landsat satellite imageries

Landsat 7 and Landsat 8 images with acquisition dates of November 06, 2010 and November 11, 2015 acquired from USGS Earth Explorer were used for this study. The data were in GeoTiff format with 16bit radiometric resolution (ranges from 0 to 65535).

#### Landsat 7

The band ratios among the first four ETM+ bands as proposed and tested in the literature were computed [[28], [29], [30], [31], [32], [33], [34]]. In the regression models established, the logarithmically transformed chlorophyll-*a* concentration was used as a dependent variable [35]. The three types of independent variables were tested: reflectance of a single band, logarithmically transformed band ratios, and ratios of logarithmically transformed single band. R2 values were computed. From the best results, a map was generated showing the chlorophyll-*a* distribution and concentration in Lagos Lagoon.

#### Conversion of Landsat 8 DN values to top of atmosphere (TOA) reflectance

The Landsat 8 DN was then converted to TOA reflectance using the Landsat 8 processing toolbox of ArcGIS 10.3.

Radiometric calibration and atmospheric correction for Landsat 8 required to achieve the purpose of chlorophyll *a* concentration retrieval [36] were conducted using the ENVI software in this study. After radiometric calibration, the un-calibrated digital numbers (DN) were converted to radiance values through the formula:

(4) L_λ_ = *M_L_Q_cal_ + A_i_*

where

*L_λ_* is the top-of-atmosphere (TOA) spectral radiance,

*M_L_* is band specific multiplicative rescaling factor from the metadata,

*A_i_* is the band specific additive rescaling factor from the metadata, then the dimensionless top-of-atmosphere reflectance *ρ*TOA can be calculated as:

(5) *ρTOA* *= πL_λ_d^2^/ESUN_λ_cosƟ_s_*

Where

*L_λ_* is the spectral radiance at the sensor,

*d*^2^ is the Earth-sun distance in astronomical units.

*ESUN* is the mean solar exoatmospheric irradiance for each band and

*θ*cos is the solar zenith angle in degrees

#### Band ratio using band 4 and band 5 reflectance

The reflectance band 4 (NIR) and band 5 (MIR) were divided to correct atmospheric distortions in the images and to obtain a band ratio of the both images.

#### Estimation of chl-*a* content

The band ratio (3_4.tif) was then divided by π to obtain the chlorophyll-*a* content using the raster calculator in ArcGIS and the regression method. Finally, the FLAASH module outputs a bottom-of-atmosphere reflectance value for each pixel and an average scene visibility and water amount estimate [37]. It is worth mentioning that the image data used in this work are all processed by FLAASH atmospheric correction. This process produced a Landsat image of all individual bands with reflectance values.

## Conclusion

Chlorophy-*a* is an indicator of phytoplankton abundance and contributes signicantly to the overall primary productivity of coastal water bodies. Chlorophyll-*a* are useful in providing information for detail assessment of algal biomass and its spatial and temporal variability. This study estimates Chl-*a* concentration using laboratory and remote sensing (using Landsat ETM and OLI images) methods. The ﬂuorometric method is extensively used for the quantitative analysis of chlorophyll *a* and phaeopigments while remote sensing extraction method is extensively used for the quantitative and qualitative mapping of chlorophyll-*a*. The procedures in this study are appropriate for all levels of chlorophyll-*a* concentration in any aquatic environment. These two methods based on their details take into consideration the scientiﬁc requirements for assessing historical and current issues about water body.

## References

[1] Hestir E.L., Brando V.E., Bresciani M., Giardino C., Matta E., Villa P., Dekker A.G. (2015). Measuring freshwater aquatic ecosystems: the need for a hyperspectral global mapping satellite mission. Remote Sens. Environ..

[2] Vignolo A., Pochettino A., Cicerone D. (2006). Water quality assessment using remote sensing techniques: Medrano Creek, Argentina. J. Environ. Manage..

[3] Vandeweerd L. (2006). The State of the Marine Environment: Regional Assessment. http://www.unep.org/gpa/documents/publications/TheStateOftheMarineEnvironmentRegionalAssessments.pdf.

[4] Guzinski R., Kass S., Huber S., Bauer-Gottwein P., Jensen I.H., Naeimi V., Doubkova M., Walli A., Tottrup C. (2014). Enabling the use of earth observation data for integrated water resource management in Africa with the water observation and information system. Remote Sens..

[5] Maxwell K., Johnson G.N. (2000). Chlorophyll fluorescence—a practical guide. J. Exp. Bot..

[6] Dos Santos A.C.A., Calijuri M.C., Moraes E.M., Adorno M.A.T., Falco P.B., Carvalho D.P., Deberdt G.L.B., Benassi S.F. (2003). Comparison of three methods for chlorophyll determination: spectrophotometry and fluorimetry in samples containing pigment mixtures and spectrophotometry in samples with separate pigments through high performance liquid chromatography. Acta Limnol. Bras..

[7] Kishino M., Tanaka A., Ishizaka J. (2005). Retrieval of chlorophyll-a, colored dissolved organic matter in Tokyo Bay using ASTER data. Remote Sens. Environ..

[8] Werdell P.J., Baley S.W. (2005). An improved in-situ bio-optical data set for ocean color algorithm development and satellite data product validation. Remote Sens. Environ..

[9] Wu J.L., Ho C.R., Huang C.C., Srivastav A.L., Tzeng J.H., Lin Y.T. (2014). Hyperspectral sensing for turbid water quality monitoring in freshwater rivers: empirical relationship between reflectance and turbidity and total solids. Sensors.

[10] Chang N.B., Imen S., Vannah B. (2015). Remote sensing for monitoring surface water quality status and ecosystem state in relation to the nutrient cycle: a 40-year perspective. Crit. Rev. Environ. Sci. Technol..

[11] Harrington J.A., Schiebe F.R., Nix J.F. (1992). Remote sensing of Lake Chicot, Arkansas: monitoring suspended sediments, turbidity, and Sechi depth with Landsat MSS data. Remote Sens. Environ..

[12] Wang Y., Xia H., Fu J., Sheng G. (2004). Water quality change in reservoirs of Shenzhen, China: detection using LANDSAT/TM data. Sci. Total Environ..

[13] Holm-Hansen O., Riemann B. (1978). Chlorophyll a determination: improvements in methodology. Oikos.

[14] Herbland A., Le Bouteiller A., Raimbault P. (1985). Size structure of phytoplankton biomass in the equatorial Atlantic Ocean. Deep-Sea Res..

[15] Ostrowska M. (1990). Fluorescence “in situ” method for the determination of chlorophyll-a concentration in sea. Oceanologia.

[16] Arar E.J., Collins G.B. (1997). Method 445.0 – In Vitro Determination of Chlorophyll a and Pheophytin a in Marine and Freshwater Algae by Fluorescence.

[17] Yentsch C.S., Menzel D.W. (1963). A method for the determination of phytoplankton chlorophyll and phaeophytin by fluorescence. Deep-Sea Res..

[18] Golterman H.L. (1975). Physiological Limnology: An Approach to the Physiology of Lake Ecosystems.

[19] Center for Earth Observation (2016). How to Convert Landsat DNs to Top of Atmosphere (ToA) Reflectance. http://yceo.yale.edu/how-convert-landsat-dns-top-atmosphere-toa-reflectance.

[20] Richter R. (1996). A spatially adaptive fast atmospheric correction algorithm. Int. J. Remote Sens..

[21] Atmospheric Correction Module (2009). Atmospheric Correction Module: QUAC and FLAASH User’s Guide, August, 2009 Edition Copyright ^©^ ITT Visual Information Solutions. https://www.exelisvis.com/portals/0/pdfs/envi/Flaash_Module.pdf.

[22] Liu Y., Islam M.A., Gao J. (2003). Quantification of shallow water quality parameters by means of remote sensing. Prog. Phys. Geogr..

[23] Fernanda S.Y.W., Enner A., Thanan W.P.R., Nilton N.I., Cláudio C.F.B., Luiz H.S.R. (2015). Estimation of chlorophyll-a concentration and the trophic state of the Barra Bonita hydroelectric reservoir using OLI/Landsat-8 images. Int. J. Environ. Res. Publ. Health.

[24] Gitelson A.A., Gritz Y., Merzlyak M.N. (2003). Relationships between leaf chlorophyll content and spectral reflectance and algorithms for non-destructive chlorophyll assessment in higher plant leaves. J. Plant Physiol..

[25] Irish R.R. (2000). Landsat 7 Science Data User’s Handbook, Report 430-15-10-003-0. http://Itpwww.gsfc.nasa.gov/IAS/handbook/handbook_toc.html.

[26] Yang L., Huang C., Homer C.G., Wylie B.K., Coan M.J. (2003). An approach for mapping large-area impervious surface: synergistic use of Landsat-7 ETM+ and high spatial resolution imagery. Can. J. Remote Sens..

[27] Huang C., Wylie B., Yang L., Homer C., Zylstra G. (2002). Derivation of a tasselled cap transformation based on Landsat 7 at-satellite reflectance. Int. J. Remote Sens..

[28] Gitelson A.A., Yacobi Y.Z., Karnieli A., Kress N. (1996). Reflectance spectra of polluted marine waters in Haifa Bay, Southeastern Mediterranean: features and application for remote estimation of chlorophyll concentration. Isr. J. Earth Sci..

[29] Chavez P.S. (1996). Image-based atmospheric corrections-revisited and revised. Photogramm. Eng. Remote Sens..

[30] Baban S.M.J. (1997). Environmental monitoring of estuaries; estimating and mapping various environmental indicators in Breydon Water Estuary, U.K., using Landsat TM imagery. Estuar. Coast. Shelf Sci..

[31] Reddy M.A. (1997). A detailed statistical study on selection of optimum IRS LISS pixel configuration for development of water quality models. Int. J. Remote Sens..

[32] Woodruff D.L., Stumpf R.P., Scope J.A., Paerl H.W. (1999). Remote estimation of water clarity in optically complex estuarine waters. Remote Sens. Environ..

[33] Braga C.Z.F., Vianna M.L., Kjerfve B. (2003). Environmental characterization of a hypersaline coastal lagoon from Landsat-5 thematic mapper data. Int. J. Remote Sens..

[34] Jensen J.R. (2005). Introductory Digital Image Processing, A Remote Sensing Perspective.

[35] Chang K.W., Shen Y., Chen P.C. (2004). Predicting algal bloom in the Techi reservoir using Landsat TM data. Int. J. Remote Sens..

[36] Chengkun Z., Min H. (2015). Mapping chlorophyll-a concentration in Laizhou Bay using landsat 8 OLI data. E-Proceedings of the 36 IAHR World Congress.

[37] Tebbs E., Remedios J., Harper D. (2013). Remote sensing of chlorophyll-a as a measure of cyanobacterial biomass in Lake Bogoria, a hypertrophic, saline-alkaline, ﬂamingo lake, using Landsat ETM+. Remote Sensing of Environment.

[38] Watanabe F.S.Y., Alcântara E., Rodrigues T.W.P., Imai N.N., Barbosa Rotta C.C.F.L.H. (2015). Estimation of Chlorophyll-a Concentration and the Trophic State of the Barra Bonita Hydroelectric Reservoir Using OLI/Landsat-8 Images. Int. J. Environ. Res. Public Health.

